# Botryoid rhabdomyosarcoma or parasites: transnasal choledoscopy as a diagnostic tool

**DOI:** 10.1055/a-2058-8389

**Published:** 2023-04-17

**Authors:** Gül Şeker, Mesut Akarsu, Yunus Güler, Özlem Gülpınar Aydın, Safiye Günden Aktaş, Yeşim Öztürk

**Affiliations:** 1 Department of Pediatrics, Division of Pediatric Gastroenterology Hepatology and Nutrition, Dokuz Eylul University Faculty of Medicine, Izmir, Turkey; 2 Department of Gastroenterology, Dokuz Eylul University Faculty of Medicine, Izmir, Turkey; 3 Department of Basic Oncology, Dokuz Eylul University Institute of Health Sciences, Izmir, Turkey

A 5-year-old girl presented with jaundice and loss of appetite. Physical examination revealed that she had a palpable liver 4 cm below the right costal arch. There were no ascites, cervical adenopathy, or splenomegaly. Laboratory values showed elevated liver enzymes and direct bilirubin.

After ultrasound and magnetic resonance cholangiopancreatography examinations, parasites and sludge were considered. Endoscopic retrograde cholangiopancreatography (ERCP) was initially performed, and when adequate patency was achieved after sphincterotomy, the procedure was continued with a transnasal scope. The first pathology report showed an epithelial fragment of the intestinal mucosa and necrobiotic material. Cholestasis improved rapidly after the procedure.

The common bile duct was cannulated with a transnasal scope, biopsies were taken, and new stents were inserted during the second ERCP. Abdominal computed tomography and magnetic resonance imaging were unremarkable ( Fig. 1 , Fig. 2 ). After the second ERCP and pathology results, a diagnosis of botryoid rhabdomyosarcoma (RMS) was made ( Fig. 3 , Fig. 4 , Video 1 ).

**Fig. 1:**
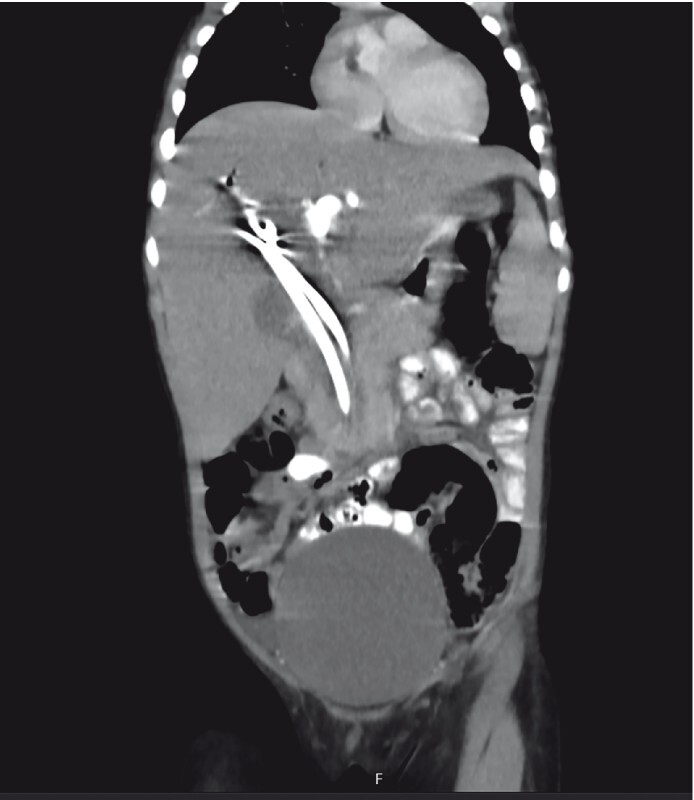
Abdominal computed tomography showed dilation of the intra- and extrahepatic bile ducts, an increase in the length of the gallbladder, and an increase in the density of the common bile duct in the distal part of the gallbladder, in a heterogeneous and occasionally tubular appearance. A parasite was considered.

**Fig. 2:**
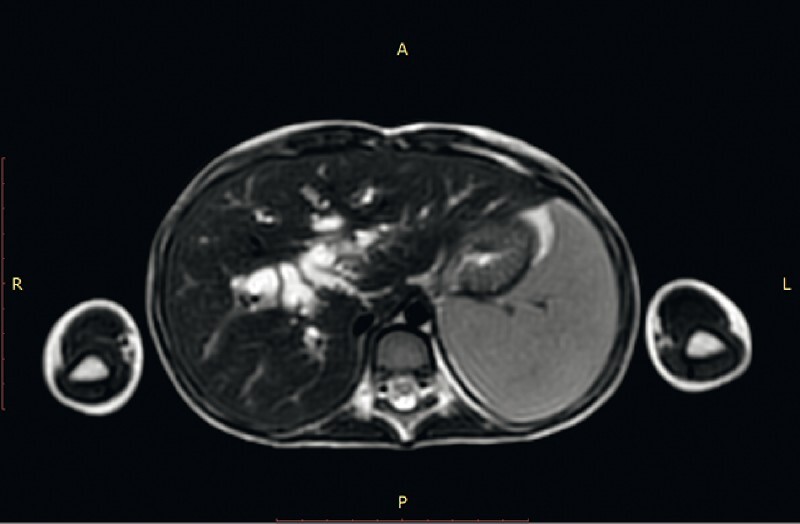
Abdominal magnetic resonance imaging. Dilation of intra- and extrahepatic bile ducts, and possible parasitic structures in the common bile duct, cystic duct, and gallbladder lumen. Patchy, subtle T2 signal enhancement near the bile ducts in the liver suggestive of cholangitis.

**Fig. 3:**
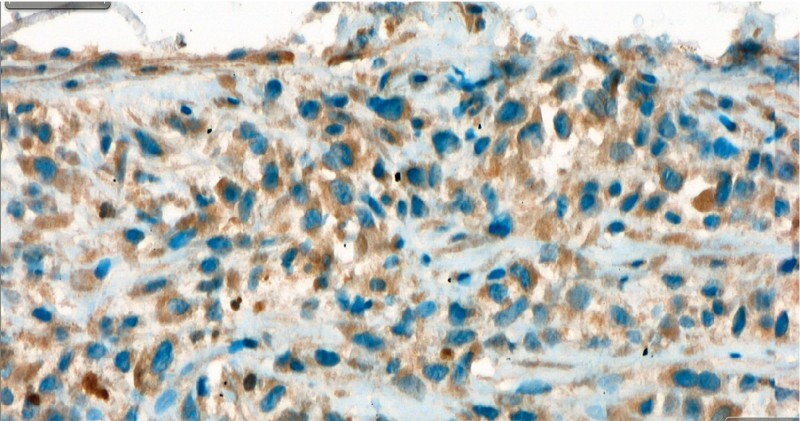
Spindle cells were immunohistochemically positive for vimentin, desmin, MyoD1, myogenin, and WT-1. These cells were negative for pancreatin, ERG, D2–40, c-kit, HMB45, LCA, CD3, S-100, and CD34. The tumor was highly vascularized.

**Fig. 4:**
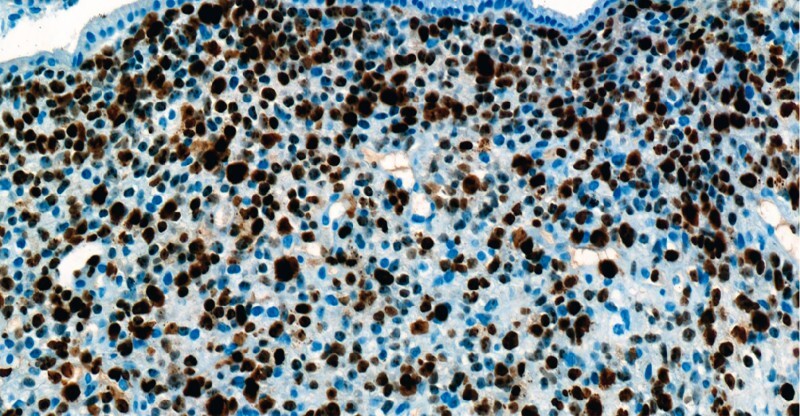
Ki-67 proliferation index was high. Based on morphology and immunohistochemistry, embryonal rhabdomyosarcoma, a botryoid variant, was diagnosed.

**Video 1** Use of endoscopic retrograde cholangiopancreatography in a pediatric case and consideration as a diagnostic tool (insertion diameter of the nasal endoscope 5.9 mm (EG-530NW; Fujinon, Tokyo, Japan).


RMS is an unusual tumor of the bile duct in children. Intermittent jaundice, fever, and anorexia are typical symptoms. The radiologic appearance of the lesion resembles that of a congenital choledochal cyst 1 2 3 . Due to its low incidence, the diagnosis of RMS is very difficult, and requires a high degree of suspicion and the use of new and appropriate imaging techniques 4 . In this case, we emphasize the importance of ERCP and choledoscopy in the diagnosis of rare RMS. Besides, this case highlights not only the importance of considering malignant etiology in pediatric cases of obstructive jaundice but also the need to consider ERCP as a diagnostic tool in children.

Endoscopy_UCTN_Code_CCL_1AZ_2AN

## Notes

Endoscopy E-Videos

https://eref.thieme.de/e-videos
*E-Videos* is an open access online section of the journal *Endoscopy* , reporting on interesting cases and new techniques in gastroenterological endoscopy. All papers include a high-quality video and are published with a Creative Commons CC-BY license. Endoscopy E-Videos qualify for HINARI discounts and waivers and eligibility is automatically checked during the submission process. We grant 100% waivers to articles whose corresponding authors are based in Group A countries and 50% waivers to those who are based in Group B countries as classified by Research4Life (see: https://www.research4life.org/access/eligibility/ ). This section has its own submission website at https://mc.manuscriptcentral.com/e-videos
